# Supplementary material for: Identification and functional analysis of non-coding regulatory small RNA FenSr3 in Bacillus amyloliquefaciens LPB-18
Source: PeerJ. 2023 May 15;11:e15236. doi: 10.7717/peerj.15236 (PMC10194069; doi:10.7717/peerj.15236)
Supplement: Supplemental Information 4 [file peerj-11-15236-s004.zip › KO/CK-vs-T1_map/map04914.html]

KEGG PATHWAY: Progesterone-mediated oocyte maturation - Reference pathway


|  |  |
| --- | --- |
| **Progesterone-mediated oocyte maturation - Reference pathway** |  |

[
Pathway menu
| Organism menu
| Pathway entry
| Show description
| User data mapping
]

Xenopus oocytes are naturally arrested at G2 of meiosis I. Exposure to either insulin/IGF-1 or the steroid hormone progesterone breaks this arrest and induces resumption of the two meiotic division cycles and maturation of the oocyte into a mature, fertilizable egg. This process is termed oocyte maturation. The transition is accompanied by an increase in maturation promoting factor (MPF or Cdc2/cyclin B) which precedes germinal vesicle breakdown (GVBD). Most reports point towards the Mos-MEK1-ERK2 pathway [where ERK is an extracellular signal-related protein kinase, MEK is a MAPK/ERK kinase and Mos is a p42(MAPK) activator] and the polo-like kinase/CDC25 pathway as responsible for the activation of MPF in meiosis, most likely triggered by a decrease in cAMP.

|  |  |  |
| --- | --- | --- |
| Reference pathway | 184% 150% 122% 100% 82% 67% 55% | 图片下载 |
